# Determining trends of respiratory tract infections in a long-term care facility pilot surveillance project

**DOI:** 10.1017/ash.2023.245

**Published:** 2023-09-29

**Authors:** Cullen Adre, Dipen Patel, Vicky Reed, Srilakshmi Velrajan, Christopher Evans, Christopher Wilson

## Abstract

**Background:** Respiratory tract infections (RTIs) in long-term care facilities (LTCFs) are particularly burdensome among residents, the COVID-19 pandemic highlighted the devastating consequences of RTIs in LTCFs. This situation has prompted the need for LTCFs to have a robust, active surveillance system to assist LTCFs with RTI identification. Such a system could assist with faster implementation of appropriate antimicrobial therapy and critical infection prevention and control. The TN Emerging Infections Program worked with CDC EIP to implement a pilot project to test the feasibility of performing RTI surveillance to inform future changes to NHSN. **Methods:** We recruited 6 LTCFs to collect prospective RTI surveillance for 6 consecutive months from October 2021 through March 2022. Data were collected for all residents meeting the RTI surveillance definitions: pneumonia, lower respiratory tract infection, influenza-like illness (including influenza), and COVID-19. These data were entered by facility workers into a REDCap database with a prospective RTI LTCF event form. Monthly data collection summaries were submitted using a designated denominator form. Descriptive statistics were used to analyze RTI data, and analyses were performed using SAS version 9.4 software. **Results:** In total, 6 facilities participated in the pilot project during the capture period. The total number of RTI cases across all facilities was 195. December had the most cases (n = 50). The most common first triggers were new RTI signs or symptoms (67.69%), laboratory results (17.44%), imaging findings (6.67%), and clinician-diagnosed RTI (8.21%). The most reported symptom was new or increased cough (57.44%). Chest radiographs were performed for 50.77% of patients. Positive viral laboratory test results were documented 29.74% of the time. Antibiotic treatments were given to 70.77% of residents. The most commonly prescribed antibiotics were cephalosporins (22.56%), macrolides (17.95%), fluoroquinolones (12.31%), and doxycycline (9.23%). Also, 17.4% of cases with antibiotic regimens had cephalosporins as monotherapy. Vaccine documentation was as follows: influenza 2020–2021 (40.51%), influenza 2021–2022 (64.1%), complete COVID-19 vaccine series (82.56%), PPSV-23 vaccine (33.85%), and PCV-13 (23.59%). **Conclusions:** RTI surveillance was incorporated smoothly into the daily workflow for facilities; the biggest barrier to effective implementation was staff turnover. A scheduled weekly time to collect data and fill out forms proved most effective. A high percentage of cases was treated with cephalosporins as monotherapy, which, based on the latest guidelines, may be suboptimal. Individual reports were sent back to facilities with a comparison to the aggregated data. These data will be used to evaluate antibiotic appropriateness and to guide future RTI surveillance efforts in the LTCF setting.

**Disclosures:** None

